# Prognostic Significance of Tumor Location in T2 Gallbladder Cancer: A Systematic Review and Meta-Analysis

**DOI:** 10.3390/jcm10153317

**Published:** 2021-07-28

**Authors:** Hyun Kang, Yoo Shin Choi, Suk-Won Suh, Geunjoo Choi, Jae Hyuk Do, Hyoung-Chul Oh, Hong Jin Kim, Seung Eun Lee

**Affiliations:** 1 Department of Anesthesiology, Chung-Ang University College of Medicine, Seoul 06973, Korea; roman00@cau.ac.kr (H.K.); pistis23@cau.ac.kr (G.C.); 2 Department of Surgery, Chung-Ang University College of Medicine, Seoul 06973, Korea; choiys@cau.ac.kr (Y.S.C.); bumboy1@cau.ac.kr (S.-W.S.); 3 Division of Gastroenterology, Department of Internal Medicine, Chung-Ang University College of Medicine, Seoul 06973, Korea; jhdo@cau.ac.kr (J.H.D.); ohcgi@cau.ac.kr (H.-C.O.); 4 Division of Surgical Oncology, Department of Surgery, University of North Carolina at Chapel Hill, Chapel Hill, NC 27599, USA; kimhj@med.unc.edu

**Keywords:** gallbladder, cancer, stage, survival, prognosis, meta-analysis

## Abstract

(1) Background: The *AJCC Cancer Staging Manual, Eighth Edition*, subdivided T2 GBC into T2a and T2b. However, there still exist a lack of evidence on the prognostic significance of tumor location. The aim of the present study was to examine the existing evidence to determine the prognostic significance of tumor location of T2 gallbladder cancer (GBC) and to evaluate the optimal surgical extent according to tumor location. (2) Methods: We searched for relevant literature published in the electronic databases PubMed, MEDLINE, Web of Science, Cochrane Library, and Embase before September 2020 using search terms related to gallbladder, cancer, and stage. Data were weighted and pooled using random-effects modeling. (3) Results: Seven studies were deemed eligible for inclusion, representing a cohort of 1789 cases of resected T2 GBC. The overall survival for T2b tumor was significantly worse than that for T2a tumor (HR, 2.141; 95% confidence interval (CI), 1.140 to 4.023; I^2^ = 71.4%; P_ch_^i2^ = 0.007). The rate of lymph node metastasis was lower in the T2a group (26.6%) than in the T2b group (36.6%) (OR, 2.164; 95% CI, 1.309 to 3.575). There was no evidence of a survival difference between the patients who underwent extended cholecystectomy and simple cholecystectomy in T2a GBC (OR, 0.802; 95% CI, 0.618 to 1.042) and T2b GBC (OR, 0.820; 95% CI, 0.620 to 1.083). (4) Conclusions: Hepatic side tumor was a significant poor prognostic factor in T2 GBC. Extended cholecystectomy and simple cholecystectomy showed comparable survival outcomes in T2 GBC, and additional large-scale prospective studies are warranted to establish evidence-based treatment guidelines for T2 GBC.

## 1. Introduction

Gallbladder cancer (GBC) is the most common biliary tract malignancy and traditionally has been associated with poor prognosis due to an asymptomatic course in early stage and being diagnosed in an advanced stage. However, because of the widespread use of laparoscopic cholecystectomy, the incidental detection of early GBC has increased. Moreover, because of the wide application of routine health checkups and advancements in imaging modalities, cases of early GBC have increased, and they show by far the best prognosis—close to 100% 5-year survival rate in some series [1,2]. Compared with T1 (early), T3 and T4 (advanced) GBC, the prognosis of T2 GBC is very heterogeneous and is hard to predict. Currently, the heterogeneity of prognosis of T2 GBC has been reported to be related to its location: A T2 GBC on the peritoneal side (T2a) has a better prognosis than one on the hepatic side (T2b) [1,2,3,4,5,6,7]. Although T2a GBC has been generally thought to have better survival than T2b GBC, there still remains controversy on the superior prognosis of T2a over T2b tumors because the data were not validated through a large cohort study before publication of the *AJCC Cancer Staging Manual, Eighth Edition* [8]. Furthermore, there is a lack of studies and evidence regarding the clinicopathological differences that affect the different prognoses. The gallbladder has a unique anatomy, and this anatomy might be associated with the tumor invasion, the mode of tumor spread and finally prognosis. The hepatic side of the gallbladder is attached directly to the liver only by loose connective tissue without serosa, and it has dense arterial, venous and lymphatic communications to allow easy invasion to the liver [9]. In contrast, the peritoneal side of the gallbladder is free from the adjacent organs. This issue also raises the question of whether liver resection of a T2a tumor should be performed. Some authors recommend not performing hepatic resection for a T2a tumor [1,5], while others recommend hepatectomy for both T2a and T2b tumors [3,4,5,6,7]. Although GBC is the most common biliary tract malignancy, its incidence rate is low, especially in Western world. Therefore, performing a randomized controlled trial to confirm the prognostic implication and to determine a proper surgical strategy is impossible and meta-analysis is essential to validate the prognostic significance of tumor location of T2 GBC and to establish appropriate management according to tumor location.

The aim of this meta-analysis was to examine the existing evidence to determine the prognostic significance of tumor location of T2 GBC and to evaluate the optimal surgical extent according to tumor location.

## 2. Materials and Methods

The protocol for this review was registered in the PROSPERO network (registration number: CRD42020178206). This systematic review and meta-analysis for the impact of tumor location on a tumor progression and survival in T2 GBC was performed according to the Meta-analysis of Observational Studies in Epidemiology (MOOSE) guidelines [10] and reported according to the guidelines of Preferred Reporting Items for Systematic Reviews and Meta-Analyses (PRISMA) [11].

### 2.1. Literature Search

Two authors (CYS and SSW) independently performed database searches using Ovid-MEDLINE, Embase, Cochrane Central Register of Controlled Trials (CENTRAL) and Google Scholar in September 2020, using search terms related to gallbladder, cancer and stage. No language or date restrictions were applied. In order to identify all related articles, we scanned the reference lists of the original papers until no further related references could be found. The reference lists of the identified studies and eligible articles were also manually searched. The search strategy, which included a combination of free text, Medical Subject Headings and EMTREE terms, is described in Appendix A (Table A1 and Table A2).

### 2.2. Selection Criteria

The inclusion and exclusion criteria of this study were determined before the systematic search.

#### 2.2.1. Study Design

Peer-reviewed cohort and case-control studies including nested case-control studies were eligible for inclusion. Data from review articles, case reports, case series, letters to the editor, posters, commentaries, proceedings, laboratory science studies, and any other non-relevant studies were excluded.

#### 2.2.2. Population

Inclusion criteria were as follows: (1) patients who were pathologically diagnosed with T2 Gallbladder cancer and (2) who underwent curative resection (extended cholecystectomy or simple cholecystectomy). No restrictions were applied in terms of sex, race/ethnicity or socioeconomic status.

#### 2.2.3. Exposure and Comparison

The definition of exposure and comparison were made based on the tumor location of T2 GBC. The exposed group was T2b (tumor invades the perimuscular connective tissue on the hepatic side), and the comparison group was T2a (tumor invades the perimuscular connective tissue on the peritoneal side).

#### 2.2.4. Outcomes Measures

The primary outcome measure of this meta-analysis was overall survival after surgery. The secondary outcome measure was the rate of lymph node metastasis, perineural invasion, vascular invasion, lymphatic invasion and recurrence after surgery. Studies reporting effect size (ES) as odds ratio (OR), relative risk (RR) or hazard ratio (HR) of tumor location to these outcome measures were included. The survival rate according to the type of surgery (extended vs. simple cholecystectomy) were also compared.

### 2.3. Study Selection

Reference lists obtained as described above were imported into Endnote software (Thompson Reuters, San Francisco, CA, USA) and duplicate articles were removed. The titles and abstracts identified through the search strategy were scanned independently by two investigators.

For reports determined to be eligible based on the title or abstract, the full paper was retrieved. Potentially relevant studies chosen by at least one investigator were retrieved and evaluated in full-text versions. Articles meeting the inclusion criteria were assessed separately by two investigators (DJH and OHC), and any discrepancies were resolved through discussion. In cases where agreement could not be reached, the disputes were resolved with the help of a third investigator (KH).

### 2.4. Data Extraction

Using a standardized extraction form, the following data were extracted: study name (along with the name of the first author and year of publication); region where the study was conducted; study design; source from which subjects were selected; age of subjects; overall survival rate; the rate of lymph node metastasis, perineural invasion, vascular invasion, lymphatic invasion and recurrence after surgery and survival rate according to the type of surgery (extended vs. simple cholecystectomy); methods for controlling covariates and the confounding variables controlled for; number of cases/controls or cohort groups; and total number of participants.

If information was missing or confusing, an attempt was made to contact the study authors to obtain the relevant information. When unsuccessful, missing information was calculated if possible from the relevant data in the study.

All interrelated data from the included studies were independently extracted and entered into standardized forms by two authors (LSE and KHJ) and then cross-checked. Any discrepancy was resolved through discussion. If an agreement could not be reached, the dispute was resolved with the aid of a third investigator (KH).

### 2.5. Study Quality Assessment

The quality of the studies was independently assessed by two authors (KH and LSE) using the Risk of Bias Assessment Tool for Non-randomized Studies (RoBANS) [12]. The quality of each study was evaluated according to the following six domains: the selection of participants; confounding variables; the measurement of exposure; the blinding of the outcome assessments; incomplete outcome data; selective outcome reporting. The methodology of each study was graded as “high”, “low” or “unclear” to indicate high risk of bias, low risk of bias and unclear risk of bias. Any discrepancies were resolved through discussion. If an agreement could not be reached, the dispute was resolved with the help of a third author (GC).

### 2.6. Statistical Analysis

All statistical analyses were performed using Comprehensive Meta-Analysis version 2.0 (CMA, Englewood, NJ, USA, 2008). Two authors (KH and GC) independently input all data into the software. The hazard ratios (HR) or odds ratios (OR) and their 95% confidence intervals (CIs) were calculated for each outcome. Between-study heterogeneity was assessed using the Cochran’s Q and Higgins’s I^2^ statistics. A *p*-value of < 10 for the chi^2^ statistic or an I^2^ greater than 50% was considered as showing heterogeneity, and data were analyzed using the Mantel–Haenszel random-effects model. Otherwise, we applied the Mantel–Haenszel fixed-effects model [13].

We conducted sensitivity analyses to evaluate the influence of individual studies on the overall effect estimate by excluding one study at a time from the analysis.

Since the number of combined studies that showed substantial heterogeneity was less than 10, t-statistics (Hartung–Knapp–Sidik–Jonkman method) were used instead of the Z-test in all random effects analyses to lower the error rate [14].

We calculated the number needed to treat (NNT) using a 95% CI based on the absolute risk reduction as an estimate of the overall clinical impact of the intervention [15]. Publication bias was not estimated, since fewer than 10 studies were included.

### 2.7. Certainty of Evidence 

The evidence grade was determined using the guidelines of the GRADE (Grading of Recommendations, Assessment, Development, and Evaluation) system, which uses sequential assessment of the evidence quality that is followed by an assessment of the risk–benefit balance and a subsequent judgment on the strength of the recommendations [16].

## 3. Results

The search of Ovid-MEDLINE, Embase and the Cochrane Central Register of Controlled Trials (CENTRAL) produced 1796 studies, and 7 in manual researching. After adjusting for duplicates, 1791 studies remained. Of these, 1782 studies were discharged because it appeared that these studies were not of interest after reviewing the title and abstracts. The full texts of the remaining nine studies were reviewed in detail and two studies were excluded for the following reasons: did not report interest of outcomes [17,18].

**Figure 1:**
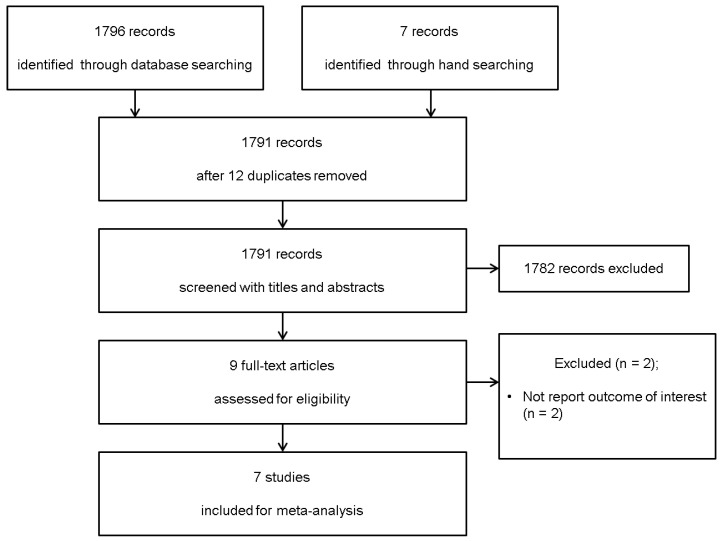
Flow diagram showing the number of abstracts and articles identified and evaluated during the review.

Thus, seven studies with a total of 1789 patients met the inclusion criteria and were finally included in this systematic review and meta-analysis (Figure 1) [1,2,3,4,5,6,7].

### 3.1. Description of the Included Studies

A description and summary of each studies’ methodology is shown in Table 1.

### 3.2. Study Quality Assessment

For all the studies included, “selection of participants” was assessed as having a high risk of bias due to their retrospective designs, and “confounding variables” was assessed as having a low risk of bias, as all major confounding variables were considered in the analysis. All the studies included were assessed as having a low risk of bias for “the measurement of exposure”, as the methods to measure exposure in their studies were regarded as “gold standard”, and as having a low risk of bias for “blinding of the outcome assessments”, as the primary outcome measure “mortality” may not be affected by whether outcome was blinded by the outcome measure. In relation to “incomplete outcome data”, two studies were assessed as having an unclear risk of bias because it was uncertain whether the incomplete outcome data could affect the study outcome. “Selective reporting” was judged as having a low risk of bias, as expected outcomes were reported in the results section in all seven studies, although no studies referenced a published protocol with pre-defined outcomes.

### 3.3. Long-Term Survival

**Figure 2:**
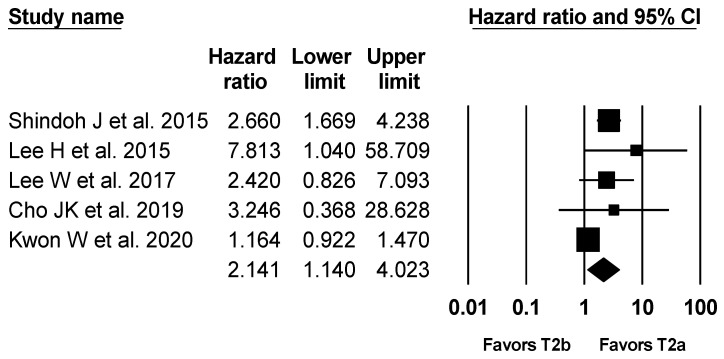
Forest plot of overall survival in resected T2a and T2b GBC patients. The figure depicts individual trials as filled squares with relative sample size and the 95% confidence interval (CI) of the difference as a solid line. The diamond shape indicates the pooled estimate and uncertainty for the combined effect.

The overall survival rate was compared using the hazard ratio in four studies [1,2,3,5]. For one study, which did not report hazard ratio for overall survival [7], we contacted the author and could obtain the data. The lower limit and upper limit of the 95% confidence interval around the hazard ratio was asymmetric after log transformation in the study of Lee W et al. [5]; therefore, we computed the 95% confidence interval assuming symmetry while maintaining width of 95% confidence interval. The combined results showed that the overall survival for T2b was significantly lower than that for T2a (hazard ratio (HR), 2.141; 95% confidence interval (CI), 1.140 to 4.023; I^2^ = 71.4%; P_ch_^i2^ = 0.007) (Figure 2). When conducting the sensitivity analysis excluding the study of Lee W et al. [5], the difference was still significant (HR, 2.131; 95% CI, 1.015 to 4.474; I^2^ = 77.0%; P_ch_^i2^ = 0.005).

### 3.4. Simple Cholecystectomy versus Extended Cholecystectomy

**Figure 3:**
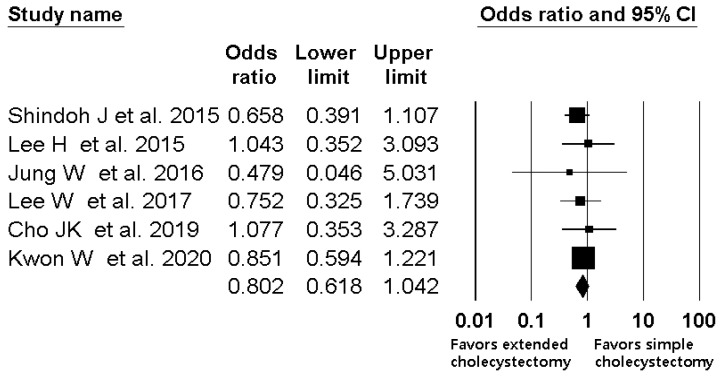
Forest plot of overall survival between extended cholecystectomy and simple cholecystectomy in T2a GBC patients. The figure depicts individual trials as filled squares with relative sample size and the 95% confidence interval (CI) of the difference as a solid line. The diamond shape indicates the pooled estimate and uncertainty for the combined effect.

A total five studies with 347 patients compared the 5-year survival rate in the T2a group [1,2,3,4,5]. For one study that did not report a 5-year survival rate in the T2a group [2], we contacted the author and obtained the data. The combined results showed no evidence of a difference (OR, 0.802; 95% CI, 0.618 to 1.042; I^2^ = 0.0%; P_ch_^i2^ = 0.928) between extended cholecystectomy and simple cholecystectomy (Figure 3), but there was a statistically significant difference in the number needed to treat (NNT, 7.5; 95% CI NNT 4.8 to NNT 17.2).

**Figure 4:**
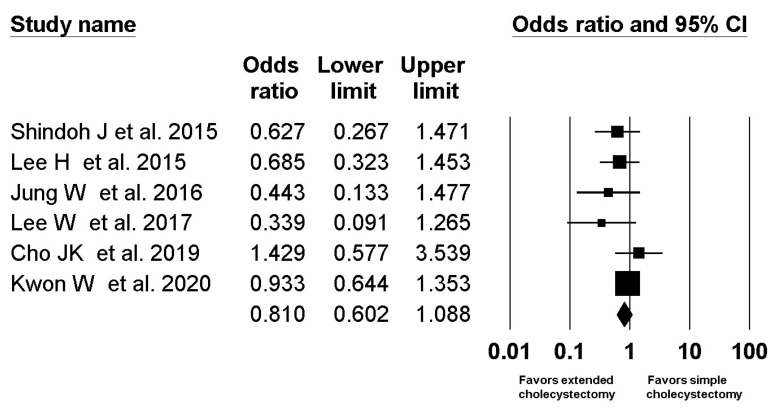
Forest plot of overall survival between extended cholecystectomy and simple cholecystectomy in T2b GBC patients. The figure depicts individual trials as filled squares with relative sample size and the 95% confidence interval (CI) of the difference as a solid line. The diamond shape indicates the pooled estimate and uncertainty for the combined effect.

A total 5 studies with 459 patients compared 5-year the survival rate in the T2b group [1,2,3,4,5]. For one study that did not report a 5-year survival rate in the T2b group [7], we contacted the author and obtained the data. The combined results showed no evidence of a difference (OR, 0.820; 95% CI, 0.620 to 1.083; I^2^ = 4.5%; P_ch_^i2^ = 0.388) between extended cholecystectomy (76.6%) and simple cholecystectomy (80.6%) (Figure 4), but there was a statistically significant difference in the number needed to treat (NNT, 15.2; 95% CI NNT 8.3 to NNT 87.0).

### 3.5. Lymph Node Metastasis

**Figure 5:**
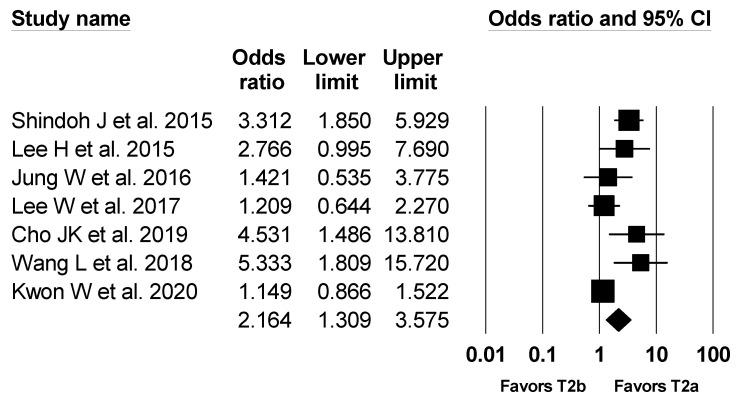
Forest plot of incidence of lymph node metastasis in T2a and T2b GBC patients. The figure depicts individual trials as filled squares with relative sample size and the 95% confidence interval (CI) of the difference as a solid line. The diamond shape indicates the pooled estimate and uncertainty for the combined effect.

A total seven studies reported lymph node metastasis [1,2,3,4,5,6,7]. Of the 1728 patients, lymph node metastasis occurred in 539 patients (31.2%). The rate of lymph node metastasis was lower in the T2a group (26.6%) compared with that in the T2b group (36.6%) (Odds ratio (OR), 2.164; 95% CI, 1.309 to 3.575; number needed to treat (NNT), 3.6; 95% CI, NNT 3.1 to NNT 4.3). However, results of the Q test and I^2^ statistics suggested substantial heterogeneity (P_chi_^2^ = 0.002, I^2^ = 71.3%). Thus, sensitivity analysis was performed by excluding one study at a time, which showed no change in statistical significance (Figure 5).

### 3.6. Perineural Invasion

**Figure 6:**
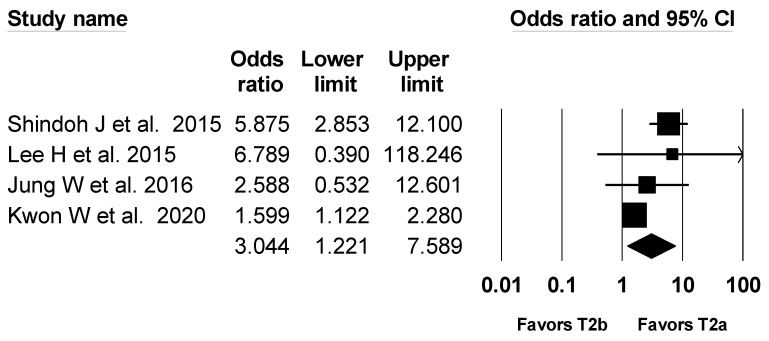
Forest plot of incidence of perineural invasion in T2a and T2b GBC patients. The figure depicts individual trials as filled squares with relative sample size and the 95% confidence interval (CI) of the difference as a solid line. The diamond shape indicates the pooled estimate and uncertainty for the combined effect.

A total four studies with 689 patients reported perineural invasion [1,3,4,5]. For one study that did not report perineural invasion [2], we contacted the author and obtained the data. The perineural invasion occurred in 277 patients among 1304 patients (21.2%). The combined results showed no evidence of a difference (OR, 2.068; 95% CI, 0.871 to 4.912; I^2^ = 77.9%; P_ch_^i2^ = 0.001) between the T2a group (18.2%) and the T2b group (24.2%), but there was a statistically significant difference in the number needed to treat (NNT, 10.3; 95% CI NNT 6.6 to NNT 23.4). Performing sensitivity analysis with removing Lee W et al. [5], heterogeneity was decreased and perineural invasion became significantly lower in the T2a group than in the T2b group (OR, 3.044; 95% CI, 1.221 to 7.589; I^2^ = 72.1%; P_ch_^i2^ = 0.013; NNT, 7.8; 95% CI NNT 5.3 to NNT 14.7) (Figure 6). 

### 3.7. Vascular Invasion

A total four studies with 689 patients reported vascular invasion [1,3,4,5]. For one study that did not report vascular invasion [7], we contacted the author and obtained the data. The vascular invasion occurred in 236 patients among 1180 patients (20.0%). The combined results showed no evidence of a difference (OR, 1.529; 95% CI, 0.782 to 2.990; I^2^ = 73.4%; P_ch_^i2^ = 0.005) between the T2a group (17.0%) and the T2b group (23.0%), but there was a statistically significant difference in the number needed to treat (NNT, 16.6; 95% CI NNT 9.5 to NNT 67.2). Sensitivity analysis by excluding one study at a time did not change statistical significance.

### 3.8. Lymphatic Invasion

A total three studies with 437 patients reported vascular invasion [1,4,5]. For one study that did not report lymphatic invasion [7], we contacted the author and obtained the data. The lymphatic invasion occurred in 307 patients among 1068 patients (28.7%). The combined results showed no evidence of a difference (OR, 1.145; 95% CI, 0.650 to 2.017; I^2^ = 59.3%; P_ch_^i2^ = 0.061; number needed to harm (NNTH), 30.0; 95% CI NNTH 7.9 to ∞ to NNTH 11.4) between the T2a group (27.0%) and the T2b group (30.3%).

### 3.9. Recurrence Rate

**Figure 7:**
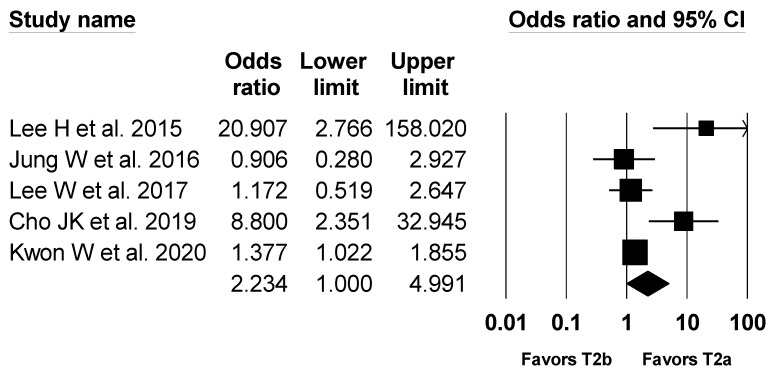
Forest plot of incidence of recurrence in T2a and T2b GBC patients. The figure depicts individual trials as filled squares with relative sample size and the 95% confidence interval (CI) of the difference as a solid line. The diamond shape indicates the pooled estimate and uncertainty for the combined effect.

A total five studies with 1394 patients reported recurrence rate [1,2,4,5,7]. The recurrence occurred in 356 patients (25.5%). The combined results showed a marginally significant difference (OR, 2.234; 95% CI, 1.000 to 4.991; I^2^ = 73.0%; P_ch_^i2^ = 0.005) between the T2a group (20.8%) and the T2b group (30.0%) (Figure 7), but there was a statistically significant difference in the number needed to treat (NNT, 3.4; 95% CI NNT 2.8 to NNT 4.3). Sensitivity analysis by excluding one study at a time did not change statistical significance.

## 4. Discussion

We conducted a systematic review and meta-analysis comparing the prognostic significance of tumor location in T2 GBC. This study is important because it is the first meta-analysis to evaluate the prognostic significance of tumor location of T2 GBC given the background of the low incidence of this disease preventing well-designed randomized trials. This meta-analysis showed that hepatic side tumor in T2 GBC (T2b) is significant poor prognostic factor. T2b GBC was strongly correlated with a higher incidence of lymph node metastasis and perineural invasion and was marginally correlated with a higher rate of recurrence, resulting in worse survival compared with the survival of the patients with peritoneal side tumor (T2a). However, extended cholecystectomy did not demonstrate survival benefit compared with simple cholecystectomy in either T2a or T2b GBC.

In spite of its limited depth of invasion into the gallbladder wall, T2 GBC generally has a high rate of LN metastasis of up to 50% [9,19,20,21]. All the studies included in the present meta-analysis showed that LN metastasis was a significant prognostic factor in multivariate analysis, and they also demonstrated that a T2b tumor has a higher possibility of LN metastasis than a T2a tumor. Therefore, a putative cause of worse prognosis of T2b tumors is thought to be the higher incidence of lymph node metastasis in T2b tumors. However, it is not known why patients with pT2b tumors tend to have a higher incidence of lymph node metastasis. Anatomical differences between the hepatic side and peritoneal side of gallbladder may be partly associated with higher rate of lymph node metastasis in T2b GBC. Perimuscular connective tissue usually contains more and larger lymphatics than the shallower layers in the normal gallbladder [22], and the wall on the hepatic side of a normal gallbladder contains more lymphatics than that on the peritoneal side [23]. The presence of more lymphatic vessels on the hepatic side can increase the likelihood that cancer cells will invade the lymphatic vessels and finally result in lymph node metastases. Further study should be performed to evaluate why the rate of lymph node metastasis in T2b GBC is higher than that of T2a GBC, and LN dissection should be performed for the purpose of treatment and prediction of prognosis in T2 GBC.

Considering the high rate of lymph node metastasis and recurrence in T2 GBC, T2 GBC may benefit from adjuvant treatment. However, it is still controversial with the indication of adjuvant treatment after surgery and standard adjuvant treatment for GBC because evidence for the role of any adjuvant therapy in GBC is insufficient. A recently published meta-analysis showed adjuvant chemoradiotherapy is associated with improved survival for the patients with resected GBC [24]. The randomized controlled BILCAP trial suggests that adjuvant capecitabine chemotherapy improves survival after biliary tract cancer resection including GBC [25]. A Korean national database study also showed that adjuvant chemotherapy significantly improved overall survival for the patients with LN metastasis in T2 GBC irrespective of regimen [26]. Therefore, the resection alone might be insufficient for GBC, and further adjuvant treatment should be considered.

In the present meta-analysis, extended cholecystectomy did not demonstrate survival benefit compared with simple cholecystectomy in either T2a or T2b GBC. This is contradictory to the current global treatment guidelines for T2 GBC that recommend extended cholecystectomy [27,28,29,30]. However, the result does not also mean the superiority of simple cholecystectomy over extended cholecystectomy. Figure 3 and Figure 4 show that most included studies had a trend of better survival after extended cholecystectomy without statistical significance. Considering the selection bias inherent in a retrospective study, the results should be interpreted cautiously and a further well-designed prospective large-scale study or randomized prospective study with very strict selection criteria should be performed.

The current meta-analysis has several limitations. First, the main limitation is that all the included studies were retrospective in nature and selection bias and treatment variations should be considered. For surgical procedures, there was no standardized operation. In some studies, simple cholecystectomy meant only cholecystectomy, while in the other studies it meant cholecystectomy with lymph node dissection. For lymph node dissection, there was no standardized extent of lymph node dissection or number of harvested lymph nodes. Additionally, because a relatively small number of patients was enrolled in each study, sub-analysis for surgical procedures according to other prognostic factors was not performed. For adjuvant treatment, there was no mention of standard methods or indication and so on. Second, the methodology to define tumor location in the included studies was not standardized, so it was unclear as to whether it was determined radiologically or pathologically or radio-pathologically, and there has been no consensus on how to classify a tumor located in the gray zone adjacent to a cystic duct, which may lead to misinterpretation of the results. Finally, most of the studies included in this analysis were from Eastern countries—Korea, Japan and China—with lack of data from Western countries. Even considering that the incidence of GBC is particularly high in Asians, most of the included studies in this meta-analysis were conducted in Asia (five out of seven studies). Considering these limitations, further prospective studies with a larger number of subjects from Eastern and Western countries using standardized treatment protocols are warranted to determine the appropriate surgical strategy for T2 GBC.

Despite these limitations, our study has demonstrated a better prognosis for T2a GBC than that of T2b GBC through the application of rigorous methodologies to provide the first systematic review investigating the prognostic significance of tumor location in T2 GBC. Since this condition is relatively rare and randomized controlled trials would not be feasible, a meta-analysis of retrospective studies is likely to be the best available evidence for rare cancers such as GBC.

## 5. Conclusions

In conclusion, a peritoneal side tumor (T2a) was significantly associated with a better prognosis than a hepatic side tumor (T2b) in T2 GBC patients. Extended cholecystectomy and simple cholecystectomy showed comparable survival outcomes in T2 GBC. Additional large-scale prospective studies using standardized methodology to define tumor location and a standardized reporting system on surgical methodology are warranted to establish evidence-based treatment guidelines for T2 GBC.

## Figures and Tables

**Table 1:** Descriptive summary of included 7 studies.

	Lee, et al. [1] (2015)		Shindoh, et al. [3] (2015)		Jung, et al. [4] (2016)		Lee, et al. [5] (2017)		Wang, et al. [6] (2018)		Cho, et al. [2] (2019)		Kwon, et al. [7] (2020)	
	T2a	T2b	T2a	T2b	T2a	T2b	T2a	T2b	T2a	T2b	T2a	T2b	T2a	T2b
No. participants	157		252		88		192		82		81		937	
	33	124	153	99	26	62	99	93	46	36	36	45	492	384
Sex (M:F)	1:1.6		1:2.3		1:1.4		1:1.4		1:1.8		1:0.9		1:1.5	
Age (yr, range)	62 (37–83)		63 (30–88)		65		NA		60 (24–96)		69 (36–88)		66 (26–91)	
Defining location	operative finding and radiological		histopathological		radiological and histopathological		radiological		histopathological		radiological		radiological and histopathological	
Operation														
Ext. Cx.	24	98			23	49	82	80			20	24	390	293
Simple Cx.	9	26			3	13	11	19			16	21	102	91
Median follow-up (mo)	40		58.9		66.5		NA		39		NA		46	
LN metastasis (%)	15	33	17	40	32	38.9	26.3	30.1	13.0	44.4	13.9	42.8	32.3	35.3
Lymphatic invasion (%)	12	19	NA		30.8	30.6	26.3	17.2	NA		NA		NA	
Perineural invasion (%)	0	9	8	33	7.7	17.7	14.1	9.7	NA		NA		NA	
Vascular invasion (%)	6	6	19	51	30.8	30.6	21.1	10.8	NA		NA		NA	
5-year overall survival (%)														
	96	62.7	64.7	42.6	64.5	65.2	84.9	71.8	60	40	96.6	76	74.5	65.5
Ext. Cx.	96	67.5	75.5	48.2	68.5	70.1	70.5	80.3	NA	NA	94.1	70.9	76.5	68.2
Simple Cx.	100	44.5	49.8	28.9	33.3	43.7	54.8	30.0	NA	NA	100	100	66.1	56.2
Recurrence (%)	3	39.5	NA		19.2	17.7	13.1	15.1	NA		8.3	44.4	24.6	31.0

Ext. Cx.—extended cholecystectomy; Simple Cx—simple cholecystectomy; NA—not available; LN—lymph node.

## Data Availability

All data used for analyses is available within the manuscript and the original publications of the included studies.

## Appendix A

**Table A1:** MEDLINE.

1	exp gallbladder neoplasm/
2	exp Gallbladder/
3	Gallbladder.mp.
4	GB.mp.
5	or/2–4
6	exp neoplasm/
7	neoplasm$.mp.
8	cancer$.mp.
9	tumo?r.mp.
10	or/6–9
11	5 and 10
12	1 or 11
13	exp neoplasm staging/
14	TNM.mp.
15	tumo?r stag$.mp.
16	cancer stag$.mp.
17	neoplasm stag$.mp.
18	stag$ system.mp.
19	or/13–18
20	12 and 19

**Table A2:** EMBASE.

1	‘gallbladder cancer’/exp
2	‘gallbladder’/exp
3	gallbladder
4	GB
5	#2 OR #3 OR #4
6	‘neoplasm’/exp
7	‘malignant neoplasm’/exp
8	cancer
9	tumor
10	#6 OR #7 OR #8 OR #9
11	#5 AND #10
12	#1 OR #11
13	‘cancer staging’/exp
14	tnm
15	tumor stag$
16	cancer stag$
17	neoplasm stag$
18	stag$ system
19	#13 OR #14 OR #15 OR #16 OR #17 OR #18
20	#12 AND #19
